# The Effect of Autologous Blood Transfusion in Open Heart Procedures on the Requirement of Allogeneic Blood Products

**DOI:** 10.7759/cureus.88742

**Published:** 2025-07-25

**Authors:** Vikas Deep Goyal, Amit Varshney, Akhilesh Pahade

**Affiliations:** 1 Surgery, Shri Ram Murti Smarak Institute of Medical Sciences, Bareilly, IND; 2 Internal Medicine, Shri Ram Murti Smarak Institute of Medical Sciences, Bareilly, IND; 3 Anesthesiology, Shri Ram Murti Smarak Institute of Medical Sciences, Bareilly, IND

**Keywords:** allogeneic blood products, autologous blood transfusion, cardiopulmonary bypass, fresh frozen plasma, open heart surgery, packed red blood cells

## Abstract

Objectives

The aim of the study was to evaluate the effect of autologous blood transfusion on the requirement of allogeneic blood products and the change in hemoglobin levels in open heart procedures.

Patients and methods

The study included 59 patients who underwent open heart procedures from July 2020 to August 2022. Group 1 (35 patients) received an autologous blood transfusion, and group 2 (24 patients) did not receive an autologous transfusion. The requirement of allogeneic blood components and other demographic and clinical parameters were compared between the two groups.

Results

In the autologous group, 22 (62.9%) patients underwent valve replacement, 9 (25.7%) patients underwent on-pump coronary artery bypass grafting, and 4 (11.4%) patients other procedures (atrial septal defect closure, pulmonary stenosis), whereas in the non-autologous group, 19 (79.7%) patients underwent valve replacement, 3 (12.5%) patients underwent on-pump coronary artery bypass, and 2 (8.33%) patients underwent other on pump procedures. The comparison of the change in hemoglobin levels after surgery between the two groups was statistically significant, with greater change seen in the autologous group. The requirement for blood products was more in group 2, but the difference was not statistically significant.

Conclusion

Use of autologous blood during cardiac surgery on pump did not reveal any significant advantage. On the contrary, change in hemoglobin from preoperative to postoperative levels was greater in the autologous group as compared to the non-autologous group.

## Introduction

Autologous blood transfusion is performed more frequently in cardiac surgery and less frequently in other specialties [1-3]. The autologous transfusion can be either preoperative blood donation (PABD), perioperative acute normovolemic hemodilution (ANH), or intraoperative or postoperative cell salvage [4,5]. Both PABD and ANH techniques of autologous blood collection and transfusion have been associated with good results. ANH is a technique practiced worldwide and involves withdrawal and reinfusion of a patient’s own blood in the perioperative period [4,5]. Many studies support the use of autologous transfusion, although some studies have reported inconclusive or insignificant difference in patient outcome [6,7]. Reducing reliance on allogeneic transfusions significantly reduces transfusion-related risks. Most of the current evidence on autologous transfusion practices in cardiac surgery originates from Western populations. There is paucity of data from resource-limited or non-Western settings, where blood component availability, clinical practices, unavailability of point-of-care testing, and transfusion triggers may vary. This study aims to fill that gap by assessing clinical outcomes in an Indian population within a tertiary care hospital setting. In this study, we analyzed the effects of autologous blood transfusion on the requirement of allogeneic blood products in the intraoperative as well as postoperative period. We hypothesized that autologous blood transfusion using the ANH technique would reduce the requirement for allogeneic blood products and be safe for patients undergoing open heart surgery. The primary objective was to assess the impact of autologous transfusion on the use of allogeneic blood products. The secondary objective was to evaluate the change in hemoglobin levels and the safety of this approach.

## Materials and methods

This retrospective study includes patients who underwent open heart procedures over a period of two years from July 2020 to August 2022. Patients receiving autologous blood transfusions were compared with those who did not receive an autologous transfusion. The ANH type of autologous transfusion was used in this study. The requirement of allogeneic blood components, hemoglobin levels, and other demographic and clinical parameters were compared between the two groups. Allocation of patients into autologous and non-autologous transfusion groups was non-randomized and based on surgeon discretion, patient suitability/hemodynamic stability, and feasibility of performing ANH. Patient-related variables were collected from the hospital database. Preoperative hemoglobin levels were recorded within 24 hours prior to surgery, while postoperative hemoglobin levels were measured within 24 hours following the surgical procedure, typically in the intensive care unit. All patients included in the study had complete datasets for the primary variables of interest. No patients were excluded post hoc, and no missing data were encountered for hemoglobin levels, transfusion details, or postoperative complications. Permission from the ethical committee of the institute was taken for the study.

Patient selection

Inclusion Criteria

The study included adult patients undergoing open heart surgeries, patients undergoing on-pump procedures, and patients undergoing elective cardiac surgeries.

Exclusion Criteria

The study excluded patients with hemoglobin levels below 10 gm/dL and patients with preoperative coagulopathies, sepsis, and multi-organ dysfunction.

Preoperative preparation

Four units of packed red blood cells (PRBCs), four units of fresh frozen plasma (FFPs), and four units of random donor platelets (RDPs) were arranged preoperatively in all the patients. Anticoagulants and antiplatelets were stopped at least three days before surgery in cases of valvular heart disease and coronary artery disease. Patients who were chronic smokers and alcoholics were preoperatively counselled for abstinence for at least 15 days and preferably one month before admission.

Operative technique

All the surgeries were performed by the same surgical team. The ANH technique was followed while collecting the autologous blood. Around 7-10 mL/kg of autologous blood was taken depending upon the body surface area and the preoperative hemoglobin levels (maximum 700 mL) and replaced with an equal volume of crystalloids before induction of anesthesia in the autologous group. The median sternotomy approach was used in all the patients. Coronary artery bypass grafting (CABG) cases were performed using the on-pump beating heart technique. Grafts used were left internal mammary artery and reversed saphenous vein grafts. Valve replacements were performed with tilting disc mechanical valve (TTK Chitra, Thiruvananthapuram, Kerala, India) using pledgeted Ethibond sutures (Ethicon Inc., Somerville, NJ, USA). During mitral valve replacement (MVR), both the anterior mitral leaflet (AML) and posterior mitral leaflet (PML) were preserved or a part of AML with PML was preserved. Divided AML was transposed to the PML position and fixed with valve sutures. Atrial septal defect (ASD) closures were performed using a pericardial patch. The autologous blood was transfused after coming off bypass and after the reversal of heparin with protamine. Patients undergoing CABG or ASD closure were operated under normothermia or mild hypothermia, whereas in cases of valve replacements, moderate hypothermia of 28-30°C was maintained during cardiopulmonary bypass (CPB).

Postoperative care

The majority of the patients were ventilated overnight. Allogeneic FFPs, RDPs, and PRBCs were given based on laboratory, hemodynamic, and clinical parameters. The goal was to maintain hemoglobin levels above 9 gm/dL. Anticoagulation in valve replacement cases and antiplatelets in CABG cases were started on the first postoperative day after the drain became minimal. Patients were usually shifted from the intensive care unit on the second postoperative day, and the majority were discharged on the sixth or seventh postoperative day. Patients were followed at one week post-discharge, then again at the end of first month, and three monthly thereafter during the first year.

Analysis

The data were expressed as mean ± standard deviation (SD) or as frequency and percentage where applicable. SPSS Version 20 (IBM Corp., Armonk, NY, USA) was used for data analysis. Independent t-tests were used for normally distributed continuous variables, and chi-square or Fisher’s exact tests were applied for categorical variables. Normality testing was not conducted prior to applying parametric tests, and no adjustments for multiple comparisons (e.g., Bonferroni correction) were made. Blinding of outcome assessors was not feasible due to the retrospective nature of the study. These are acknowledged in the limitations. The two groups were compared for age, sex, preoperative and postoperative hemoglobin levels, change in hemoglobin levels, and number of allogeneic blood products used, complications, and hospital stay.

## Results

Demographic and clinical data from all the patients in the study are presented in tabular format (Table 1). The male-to-female ratio in the study was 1.26:1. In the autologous group, 22 (62.9%) patients underwent valve replacement, 9 (25.7%) patients underwent on-pump CABG, and 4 (11.4%) patients other procedures (ASD closure, pulmonary stenosis), whereas in the non-autologous group, 19 (79.7%) patients underwent valve replacement, 3 (12.5%) patients underwent on-pump coronary artery bypass, and 2 (8.33%) patients underwent other on pump procedures. The average requirement of the number of units of PRBC, FFP, and RDP was 2.66 ± 2.15, 2.23 ± 1.83, and 2.74± 1.7, respectively, in group 1 and 2.96 ± 1.81, 2.13 ± 1.87, and 3.79 ± 2.87, respectively, in group 2. Although the requirement for blood products was more in group 2 than in group 1, the difference was not statistically significant. Preoperative hemoglobin levels were 14.04 ± 1.11 gm/dL and 12.02 ± 1.28 gm/dL in groups 1 and 2, respectively. Postoperative hemoglobin levels were 10.59 ± 0.79 and 10.09 ± 1.05 in group 1 and group 2, respectively. Although mean preoperative and postoperative hemoglobin levels were lower in group 2, the comparison of the change in hemoglobin after surgery between the two groups was statistically significant, with greater change seen in the autologous group. There was a single mortality in group 2 due to stroke. Two patients in group 1 and three patients in group 2 required re-exploration for bleeding, but the difference was not found to be statistically significant. The mean hospital stay was 11.09 ± 3.43 days in group 1 and 10.08 ± 2.52 days in group 2.

**Table 1:** Comparison between the demographic and clinical parameters of the non-autologous and the autologous groups. *p < 0.001 (significant at 99%). **p < 0.05 (significant at 95%). CABG, coronary artery bypass grafting; Hb, hemoglobin level; PRBC, packed red blood cells; FFP, fresh frozen plasma; RDP, random donor platelets

Parameter	Non-Autologous Group (Group 2)	Autologous Group (group 1)	p-Value	Test Used for p-value Calculation
Demographics				
Number of patients	24	35	0.447	Chi-square test
Male	12 (50%)	21 (60%)		
Female	12 (50%)	14 (40%)		
Age (years)	35.25 ± 15.64	41.57 ± 12.75	0.094	t-test
Clinical data				
Valve replacement	19 (79.17%)	22 (62.9%)	0.386	Chi-Square test
CABG	3 (12.5%)	9 (25.7%)		
Other procedures	2 (8.33%)	4 (11.4%)		
Preoperative Hb (gm/dL)	12.02 ± 1.28	14.04 ± 1.11	<0.001*	t-test
Postoperative Hb (gm/dL)	10.09 ± 1.05	10.59 ± 0.79	0.04**	t-test
Change in Hb (gm/dL)	1.93 ± 1.48	3.44 ± 1.11	<0.001*	t-test
Number of PRBC units used	2.96 ± 1.81	2.66 ± 2.15	0.576	t-test
Number of FFP units used	2.13 ± 1.87	2.23 ± 1.83	0.833	t-test
Number of RDP units used	3.79 ± 2.87	2.74 ± 1.7	0.118	t-test
Re-exploration	3 (12.5%)	2 (5.7%)	0.358	Chi-square test
Hospital stay (days)	10.08 ± 2.52	11.09 ± 3.43	0.227	t-test
Mortality rate	1 (4.17%)	0 (0%)	0.223	Chi-square test

## Discussion

PRBC transfusion is associated with an increased risk of graft thrombosis after CABG, and there is also an increased incidence of stroke, deep vein thrombosis, and peripheral graft thrombosis [8]. RBC transfusion has been associated with a 20% increased hazard of graft occlusion [8]. The probable reason may be either an immune reaction or the activation of the coagulation system. Blood transfusion increases early and long-term mortality post-cardiac surgery [9]. Bhaskar et al. reported a 66% increase in mortality with transfusion [9]. Transfusing PRBC and other blood products had been reported to be an independent dose-dependent risk factor for early mortality after CABG [10]. Transfusing more than three units is associated with worse outcomes [10]. Long-term mortality is also increased with blood transfusion during or after CABG [11]. Reports in the literature also mention increased incidence of postoperative complications including mortality if older PRBC units of more than two weeks are transfused. Transfusion is not recommended if the hemoglobin level is more than 10 gm/dL [12].

The use of allogeneic blood transfusion has to be judicious as it may be associated with adverse reactions. The adverse transfusion reactions include acute (<24 hours) or delayed (> 24 hours). Acute and delayed reactions are either immunologic or non-immunologic [13]. Allergic and febrile non-hemolytic reactions are the commonest of all transfusion-related adverse effects [13]. Some studies show more adverse reactions with platelet transfusion, while some studies show it with PRBCs [13,14]. Transfusion-related acute lung injuries and transfusion-associated circulatory overload are important causes of major complications with transfusion apart from anaphylaxis and major hemolytic reactions [15]. Major transfusion is also associated with an increased risk of nosocomial infections and poor patient outcomes in critical patients [16]. Platelet transfusion is more commonly associated with transfusion-related bacteremia than transfusion of PRBCs [17].

The use of autologous blood preserves platelets and red blood cells from the adverse effects of CPB. The harmful effects of CPB include activation of the complement system, whole-body inflammatory reaction, formation of micro-emboli, and hemolysis. The risk of transfusion-associated infection is also reduced along with cost-effectiveness. Another major advantage of using autologous blood may be decreased incidence of immunologic reactions due to reduced exposure to allogeneic blood products. Disadvantages of drawing autologous blood include excessive hemodilution if the hemoglobin/hematocrit levels are low preoperatively, chances of contamination, or improper storage if improper technique is used especially when done outside the blood bank. The use of autologous transfusion using the ANH technique has been reported to be safe [18]. Mladinov et al. in their study on autologous transfusion in aortic surgeries concluded that autologous transfusion using the ANH technique is safe and significantly reduces the need for FFP and platelet requirement, although there was no difference in the requirement of PRBCs [18]. Barile et al. in their meta-analysis conclude that autologous transfusion using the ANH technique reduces the requirement of allogeneic blood transfusion and also decreases blood loss after surgery [19].

There are many studies available in the literature on the use of autologous transfusion; most of the studies have shown favorable results, and few are equivocal [19-22]. Zisman et al. evaluated the effect of autologous transfusion on coagulation dysfunction using thromboelastography and concluded that autologous transfusion did not affect post-CPB coagulopathy [22]. Mahoori et al. conducted a randomized trial to evaluate the effect of autologous transfusion in patients undergoing CABG using CPB. They concluded that autologous transfusion is safe and effective in decreasing the need for allogeneic blood products [23]. Pawaskar et al. in their meta-analysis concluded that the use of autologous transfusion in total knee arthroplasty significantly reduced the need for allogeneic blood transfusions [24], although no transfusion is the best alternative if hemodynamics and hemoglobin are in the normal range.

In this retrospective analysis, we observed greater postoperative hemoglobin drop in the autologous group, which was an unexpected finding. This contradicts the anticipated benefit associated with autologous transfusion. We believe that the possible reasons for this observation include dilutional effects of the ANH technique and variations in intraoperative fluid management. We also noted that the autologous group had significantly higher preoperative hemoglobin levels, which may have influenced both the decision to use ANH and the magnitude of postoperative hemoglobin decline. This baseline imbalance represents a possible confounding factor that may have impacted transfusion needs and affects the interpretation of group comparisons. The results should hence be interpreted as preliminary rather than definitive.

Despite these limitations, the safety profile of autologous transfusion in this cohort was favorable. In our study, except for one case, no major adverse events or transfusion reactions were observed. One of the patients in group 2 had a peri-operative stroke, the cause of which could not be accurately defined and is possibly multifactorial. The patient underwent double valve replacement and had a stroke involving the posterior circulation. The patient required re-exploration for bleeding and also required a major transfusion. The probable causes included cardiac or carotid or air emboli, major transfusion, and cerebral hypoperfusion.

Limitations

All retrospective studies, including ours, have some limitations. This includes lack of randomization, blinding, and inadequate control over confounding variables. The restricted sample size limits statistical power. In light of these limitations, the findings of this retrospective research should be considered preliminary. We aim to conduct larger prospective, randomized, and blinded trials with standard protocols to validate the observations obtained through this study.

## Conclusions

Use of autologous blood during cardiac surgery on pump did not reveal any significant advantage. In parameters such as requirement of blood components, complications, and hospital stay, the difference between the autologous and the non-autologous group was not statistically significant. On the contrary, change in hemoglobin level from preoperative to postoperative levels was greater in the autologous group as compared to the non-autologous group.
